# AI-enabled image fraud in scientific publications

**DOI:** 10.1016/j.patter.2022.100511

**Published:** 2022-07-08

**Authors:** Jinjin Gu, Xinlei Wang, Chenang Li, Junhua Zhao, Weijin Fu, Gaoqi Liang, Jing Qiu

**Affiliations:** 1 School of Electrical and Information Engineering, University of Sydney, Sydney, NSW, Australia; 2 School of Science and Engineering, The Chinese University of Hong Kong, Shenzhen, China; 3 Shenzhen Institute of Artificial Intelligence and Robotics for Society (AIRS), Shenzhen, China; 4 Department of Urology, The First Affiliated Hospital of Guangxi Medical University, Guangxi, China

## Abstract

Destroying image integrity in scientific papers may result in serious consequences. Inappropriate duplication and fabrication of images are two common misconducts in this aspect. The rapid development of artificial-intelligence technology has brought to us promising image-generation models that can produce realistic fake images. Here, we show that such advanced generative models threaten the publishing system in academia as they may be used to generate fake scientific images that cannot be effectively identified. We demonstrate the disturbing potential of these generative models in synthesizing fake images, plagiarizing existing images, and deliberately modifying images. It is very difficult to identify images generated by these models by visual inspection, image-forensic tools, and detection tools due to the unique paradigm of the generative models for processing images. This perspective reveals vast risks and arouses the vigilance of the scientific community on fake scientific images generated by artificial intelligence (AI) models.

## The bigger picture

This perspective reports on the vast risk of potential image fraud based on artificial intelligence (AI) generative technologies in academic publications that have been neglected. This article discusses the scenarios, capabilities, and effects of AI algorithms used in academic fraud. The issue described in this perspective is not only relevant to computer scientists. As members of the scientific community, each of us will be deeply involved in the peer-review process. Each of us may be deceived by the AI image-fraud methods described in this article. Although the algorithm developing itself belongs to the field of computer science, its impact, as mentioned in this perspective, is more related to a wider range of scientific fields, such as biology, medicine, and natural science. Arousing their attention to this threat is a necessary condition to resist this threat. Combined with state-of-the-art AI research, this perspective also discusses possible preventive measures to respond to this potential threat.

## Teaser

In this perspective, we report on the vast risk of potential image fraud based on artificial intelligence (AI) generative technologies in academic publications that have been neglected. We discuss the scenarios, capabilities, and effects of generative models used in academic fraud. We demonstrate this risk through visualized interdisciplinary cases and conduct a user study. Combined with state-of-the-art AI research, we also discussed possible preventive measures to respond to this potential threat.

## Introduction

Inappropriately duplicating and fabricating images in scientific papers would have serious consequences. Editors and reviewers may be deceived, scientific communities may be misled, and research resources may be wasted. To prevent this type of misconduct, people are motivated to search for efficient detection and forensic strategies. Recently, there is a high expectation that artificial intelligence (AI) may bring new techniques for automatic inspection of images fraud in academic publications. Despite controversies and difficulties, progress in this area is being made.^1^

However, the whirlwind of progress in AI has not only produced a steady stream of advanced image-retrieval and fraud-detection techniques but has also brought about promising image-editing and -generation tools.^2^, ^3^, ^4^, ^5^, ^6^, ^7^ These tools can generate images that are increasingly indistinguishable for automated checking systems and even human judgment. A successful representative of image-generation techniques is the generative adversarial network (GAN).^8^ The GAN takes the adversariness of two deep neural networks (a generator and a discriminator) as the training paradigm and then automatically generates high-fidelity images out of nothing. Advanced generative models may be potentially applied to many fields. When they are widely used, “seeing is believing” may no longer hold true.^9^

It is not news that generative models are abused to a large extent and pose a threat to society. A typical example is Deepfake,^10^ an algorithm that generates realistic fake images and videos in which a person in an existing image is replaced with someone else. News and videos produced by Deepfake can have tremendous implications. As more fields are involved, the threats brought about by these new technologies cannot be ignored. An important issue that we need to be alerted to is that intelligent generative models are used to forge images of scientific evidence and thus threaten academic integrity in publishing. Although it has not been formally reported, due to the effectiveness and easy accessibility of these advanced technologies, such forgeries, some of which are not detectable at all, may become disturbingly common.

In this perspective, we reveal how these advanced generative models might be abused for scientific image fraud with examples. We also demonstrate the identification accuracy of this fraud by both human experts and AI techniques. Our examples and identification results show troubling signs that this type of image fraud is efficient and covert and is expected to pose a threat to academic publishing. At last, we explore possible responses to this threat. We anticipate that our article will attract the scientific community’s attention and bring about discussions on this emerging issue so that better responses can be developed and implemented.

## Scientific image fraud using generated models

Although the criteria of detecting misconduct in the scientific community are not uniform, the following three situations are acknowledged as severe cases: (1) fabrication of non-existent images, (2) falsification or manipulation of existing images, and (3) plagiarism. Among the cases that have been revealed above, inappropriate duplication and editing are the most common measures to commit these misconducts.^11^ The duplication of images includes using multiple identical images to represent different experimental results, reusing or plagiarizing images in previous publications as new experimental evidence, or creating images by synthesizing existing ones using rotating, scaling, cropping, and splicing. The editing of images involves using image-processing software to modify or tamper with images to meet authors’ expectations. However, both duplication and modification would leave traces, such as repetition coincidences that are impossible to get to appear naturally or traces of modification revealed by image-forensic tools such as inverse or false-color view.

**Figure 1:**
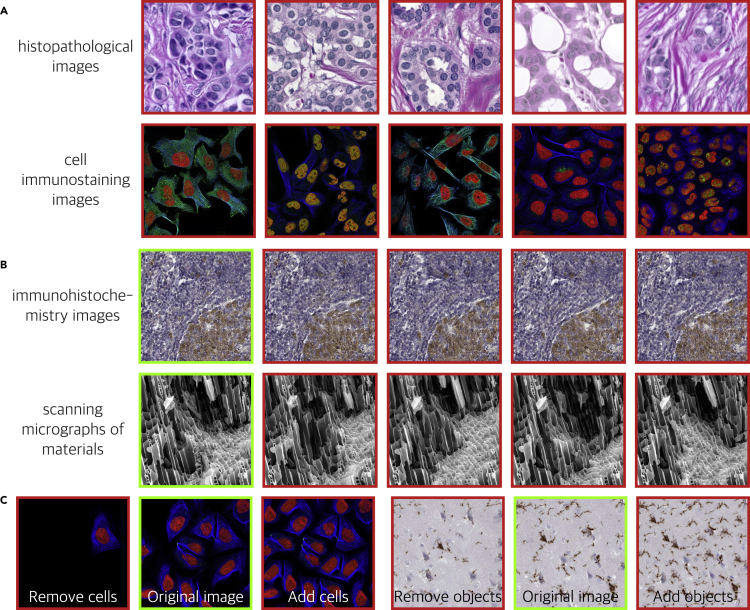
Scientific image fraud by intelligent models We show several fake images generated by generative models. The images with the red border are all computer generated, while the images with the green border are real ones. (A) Sampling fake images from a well-trained generative model. Fake images above are created by an advanced generation technology called StyleGAN.^12^ All these images are fake and meaningless in science. (B) Regenerating images using a generative model trained on a single image. For each group, the last four images are regenerated from the first real image. These images can escape the duplication detection methods based on the comparison of details because they have totally different local details. (C) Manipulating images using generative models. The generative models manipulate images by directly generating images that are similar in features but with modified content. For each group, the images in the middle are the original images, and the images on both sides are deliberately manipulated fake images.

In contrast to the above “traditional” methods, generative models generate images from scratch or regenerate existing images. The following scenarios are used to show how generative models are misused. Experienced researchers may collect many scientific images in a specific field first. The most general paradigm of generative models is to capture the underlying patterns in these scientific images and fit the distribution of the target data. Sampling using trained generative models can produce fake images that follow patterns similar to the real images. Images generated by these models are visually realistic and even scientifically self consistent (see Figure 1A). These images are meaningless in science, but one may use them as evidence to report experiments that have never been conducted. In any field where a large amount of image data can be obtained, such generated images may become a source of fake scientific images.

Different from the above cases in which the models need to be trained using a large image dataset, another novel generative paradigm allows the model to be trained with a single image. The trained model can be used for image resampling or manipulation. SinGAN is an example of this paradigm.^13^ It learns patch distribution hierarchically at different scales of an image and then regenerates high-quality, diverse images with the main style or with the content unchanged. The regenerated images preserve the statistical characteristics but have different local details compared with the original ones (see Figure 1B). This technique can be used to plagiarize published images or reuse existing images, such as reporting non-existent control-group experiments.

Apart from the fact that generated fake images may be used deliberately, modifications may also be used to produce images that meet authors’ expectations in experiments. The generative models manipulate images by directly generating images featuring similar appearances but modified content.^13^^,^^14^ For example, one may remove some cells from the image through an inpainting generative model or add new cells through an image harmonization model (see Figure 1C). In some cases, generative models are more remarkable for their ability to create images of different things that may not exist at all. The generative model may disentangle features of images during the training phase. Based on this, the model may mix these features and synthesize images that do not conform to the natural distribution of data, e.g., proteins that appear in a cell’s image where they should not have appeared.

## Risks of AI-enabled image fraud

**Figure 2:**
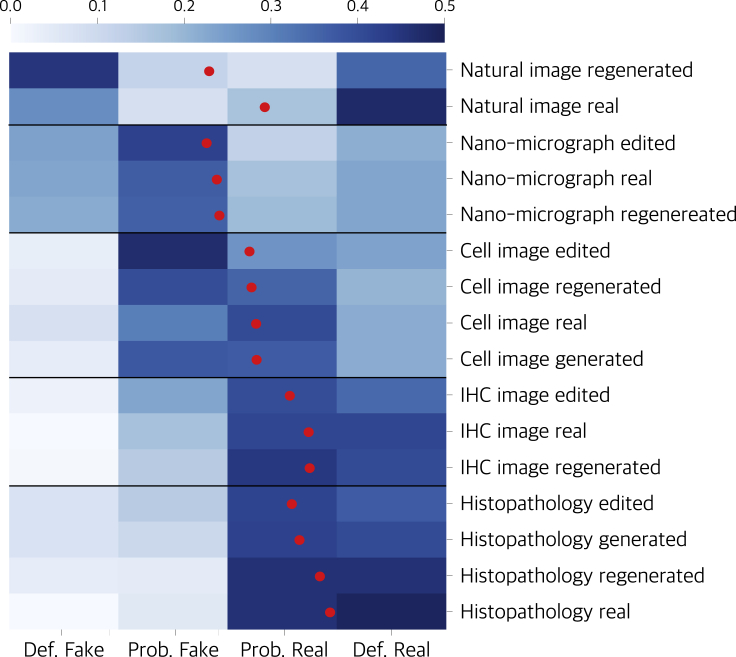
Images forged by generative models are hard to distinguish We conducted a human-opinion study. This figure shows the normalized histogram of votes per image type. The image used for evaluation consists of five categories: (1) natural images, (2) scanning micrographs of nano materials (nano-micrograph), (3) cell immunostaining images, (4) immunohistochemistry (IHC) images, and (5) histopathological images. In total, 800 images are involved, and each image is rated by at least ten medical experts. The voting scale was between 1 to 4 corresponding to the following: 1 – definitely fake, 2 – probably fake, 3 – probably real, and 4 – definitely real. Mean scores are shown as red dots.

The dangers of the fraud methods described above can be brought up in several ways, of which their difficult-to-detect nature is the most important one. Firstly, it is difficult for editors and reviewers to find such frauds through visual inspection during the peer-review process. A user study indicates that scientific images generated by generative models are likely to deceive the judgment of human experts (see Figure 2). The distribution of collected human ratings shows interesting patterns. It can be seen that humans tend to be more confident in the judgment of natural images, which is reflected in the fact that most of the ratings are either “definitely real” or “definitely fake.” For scientific images, their relatively simple image structure makes them easier to learn by generative models. The difference between the real and generated images is more subtle and imperceptible, so the average rating is biased toward “real,” and the ratings are also less confident. Secondly, the image-generation process is controlled by random noise, and different noise vectors create different images. The unnatural repetition between generated fake images no longer exists, which renders duplication inspection based on retrieving and comparing image details invalid. Third, as image generation is an end-to-end integrated process, there are no intrinsic irregularities of modification that existing image-forensic tools can detect. Detection of such generated images relies on features or fingerprints left by the generative model. This introduces very large uncertainties and difficulties for detection.

**Figure 3:**
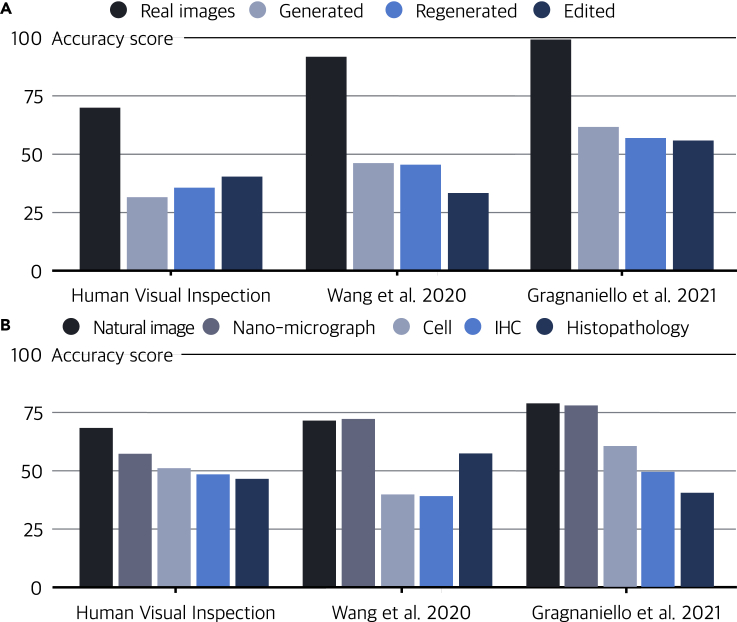
The accuracy of detecting generated images for each test subject (A) Image-fraud-detection accuracy for different fraud methods. (B) Image-fraud-detection accuracy for different image types.

In response to the threats posed by fake scientific images, research on the quality and integrity in scientific literature has attracted significant attention.^15^^,^^16^ The current forensic methods for scientific image fraud rely on unnatural repetitions found through visual inspection^11^ or intrinsic irregularities visualized through forensic tools. On the research front, AI is also expected to bring about tools for efficient automatic image-fraud detection to address the difficulty of detecting such fraud.^17^, ^18^, ^19^, ^20^ Recent studies suggest that images created by generative models may retain detectable systematic flaws that may distinguish them from authentic images.^21^, ^22^, ^23^, ^24^, ^25^ AI forensic tools can be built to tell generated images from real ones. We test two state-of-the-art AI forensic tools by using them to analyze the fake scientific images described above. We include the image classifier provided by Wang et al.,^21^ which was trained on ProGAN^6^-generated images with careful pre- and post-processing and data augmentation, and the GAN image detector proposed by Gragnaniello et al.,^26^ which was developed based on a limited sub-sampling network architecture and a contrastive-learning paradigm. The results are shown in Figures 3A and 3B. Wang et al.^21^ only achieved a similar accuracy performance to human visual inspection, and Gragnaniello et al.^26^ achieved generally better performance against Wang et al.^21^ But neither method can make good enough detections, and relying on such accuracy is not enough to mitigate the threat of image forgery based on generative models. Imperfect automated forensic tools are also highly vulnerable. A malicious user may simply select a fake image that passes the detection threshold, as a single fake image is the only thing he/she needs to achieve his/her goal. The limitation of existing methods points to the fact that the detection and forensics of scientific image fraud is still open to questions.

Another equally dangerous thing is that, unlike manually modifying or forging images with software, the cost of using these advanced models is close to negligible. For one thing, researchers are very open to the open source and sharing of these advanced generative-model technologies. The result is that all these technologies for intelligent generative models may be shared with anyone defenselessly; for example, all of the techniques involved in this article are easily available on the Internet. This greatly lowers the barrier to entry for anyone trying this type of technology, which, in turn, further gives rise to the possibility for the abuse of these technologies. For another thing, many intelligent generative models can automatically process and generate images without human intervention. Making fake scientific images no longer requires complicated human labor but can be mass produced. This has the potential to make it easier for some “paper factories”^27^ to systemically produce falsified research papers.

## The fight against AI-enabled image fraud

There is an urgent need for effective measures to respond to this potential threat. Most critically, people need first to be subjectively prepared for the new risks brought by these new technologies. Although no cases of using such intelligent image technologies have been reported, a more worrying possibility is that this kind of misconduct has quietly occurred somewhere. The problem is that it has not yet been found. Nevertheless, a window of opportunity remains open to reduce the risks to a certain extent by improving the management system or process before such a high-tech fraud pervades in scientific publications.

In terms of all preventive measures that may be taken for the moment, asking authors to provide more detailed high-resolution raw image data is the most convenient one. Although impressive progress has been made, generative models are still straggled in generating large-size high-fidelity images. The high computational resources and algorithm complexity required to generate large-size fake images will increase the threshold of such frauds. In addition, we should continue to develop forensic tools for advanced image-generation and -processing models. Tools specialized for scientific images should also be given great importance, as we see that the detection accuracy is significantly better for natural images than for scientific images. An important reason for this is that the existing tools are developed based on natural images. Although the current situation is not optimistic, the advantage of these forensic tools lies in the ability to perform large-scale automatic screening. At last, when developing new image-generation technology, we must again consider the possible social impact of such technologies and attempt to eliminate the risk of such technologies being abused as much as possible. For example, when releasing the source code of generative models that may be used for improper purposes, we may annotate generated images through encryption or steganography.

## Conclusion

Our discussion demonstrates that AI-enabled image fraud may pose serious challenges to the field of academic publishing. The difficult-to-detect nature, inexpensiveness, availability, and ease-of-use of advanced image generative models become major sources of threats when they are abused for scientific image fraud. We also explore responses to this type of fraud. However, the confrontation between new technologies and countermeasures that prevent them from being abused will become an enduring cat-and-mouse game. Perhaps when these advanced technologies are abused, our cost of obtaining the truth has been irretrievably increased.

## Appendix A: Data acquisition

In this perspective, we discussed three methods for image fraud in the main text, namely image generation, image regeneration or resampling, and image editing. The images used for evaluation may be classified into five categories: (1) natural images, such as natural sceneries, architectures, flora, and fauna; (2) scanning micrographs of nano materials collected from Internet; (3) cell immunostaining confocal microscope images from the Human Protein Atlas dataset;^28^ (4) immunohistochemistry (IHC) images collected from clinical and the Human Protein Atlas datasets;^28^ and (5) histopathological images from the breast cancer histopathological dataset (BreCaHAD).^29^

Two generative models based on StyleGAN^12^ were trained by using the cell immunostaining image dataset and the BreCaHAD histopathological image dataset. The generated images are 512 × 512 pixels. For the training of the StyleGAN generator, we follow the official suggestions. Eight NVIDIA V100 computing cards were used in the training, and the process lasted for 14 days. We used SinGAN^13^ for the image-regeneration experiments. For each image category, we selected 10 images and regenerated 5 times for each trained model. The regenerated images are 512 × 512 pixels. We follow the official suggestions of applying SinGAN. We also used the NVIDIA V100 computing card for experiments. It takes about 5 h to calculate one image. For the edited images, we also employed SinGAN. SinGAN achieves image manipulation or image harmonization by regenerating images based on a modified input image. We demonstrated adding or removing cells or objects in the images by using cell immunostaining and IHC images.

## Appendix B: User study

A total of 800 images were involved in the user study. For each image category and each image-fraud method, we prepared at least 50 images. We also prepared 50 real images for each category as a comparison. Ten volunteers with rich experience in the fields of medicine and biology participated in the study. Each volunteer was asked to fill out a set of questionnaires, and each questionnaire was limited to 16 questions. In order to prevent volunteers from feeling exhausted, the questionnaire was conducted at different times during a week. In each questionnaire, volunteers saw a set of the above images. Volunteers were informed that these images may appear in some scientific papers, popular science articles, and reports. They were also informed that these images may contain a number of unknown false, edited, or forged content. Each image may appear multiple times, and the number of times each image appeared has nothing to do with its authenticity. We asked volunteers to evaluate the authenticity of each picture based on their professional knowledge and intuition. The voting scale was between 1 and 4: 1 – definitely fake, 2 – probably fake, 3 – probably real, and 4 – definitely real. Volunteers were invited to choose the most suitable option.
